# Characterization of sex-related differences in allergen house dust mite-challenged airway inflammation, in two different strains of mice

**DOI:** 10.1038/s41598-022-25327-7

**Published:** 2022-12-02

**Authors:** Dina H. D. Mostafa, Mahadevappa Hemshekhar, Hadeesha Piyadasa, Anthony Altieri, Andrew J. Halayko, Christopher D. Pascoe, Neeloffer Mookherjee

**Affiliations:** 1 Department of Immunology, University of Manitoba, 799 JBRC, 715 McDermot Avenue, Winnipeg, MB R3E 3P4 Canada; 2 Manitoba Centre for Proteomics and Systems Biology, Department of Internal Medicine, University of Manitoba, Winnipeg, MB Canada; 3 Department of Pathology, School of Medicine, Stanford University, Palo Alto, CA 94304 USA; 4 Department of Physiology and Pathophysiology, University of Manitoba, Winnipeg, MB Canada; 5 Biology of Breathing Group, The Children’s Hospital Research Institute of Manitoba, Winnipeg, MB Canada

**Keywords:** Immunology, Inflammation, Mucosal immunology

## Abstract

Biological sex impacts disease prevalence, severity and response to therapy in asthma, however preclinical studies often use only one sex in murine models. Here, we detail sex-related differences in immune responses using a house dust mite (HDM)-challenge model of acute airway inflammation, in adult mice of two different strains (BALB/c and C57BL/6NJ). Female and male mice were challenged (intranasally) with HDM extract (~ 25 μg) for 2 weeks (N = 10 per group). Increase in serum HDM-specific IgE showed a female bias, which was statistically significant in BALB/c mice. We compared naïve and HDM-challenged mice to define immune responses in the lungs by assessing leukocyte accumulation in the bronchoalveolar lavage fluid (BALF), and profiling the abundance of 29 different cytokines in BALF and lung tissue lysates. Our results demonstrate specific sex-related and strain-dependent differences in airway inflammation. For example, HDM-driven accumulation of neutrophils, eosinophils and macrophages were significantly higher in females compared to males, in BALB/c mice. In contrast, HDM-mediated eosinophil accumulation was higher in males compared to females, in C57BL/6NJ mice. Differences in lung cytokine profiles indicated that HDM drives a T-helper (Th)17-biased response with higher IL-17 levels in female BALB/c mice compared to males, whereas female C57BL/6NJ mice elicit a mixed Th1/Th2-skewed response. Male mice of both strains showed higher levels of specific Th2-skewed cytokines, such as IL-21, IL-25 and IL-9, in response to HDM. Overall, this study details sex dimorphism in HDM-mediated airway inflammation in mice, which will be a valuable resource for preclinical studies in allergic airway inflammation and asthma.

## Introduction

Biological sex influences disease prevalence, severity, and response to therapy in respiratory diseases characterized by airway inflammation e.g. asthma. In children and early adolescents, asthma is more prevalent in boys, but this changes after puberty^1,2^. Adult females predominantly exhibit higher asthma prevalence and severity compared to males^3^. Various epidemiological and clinical studies also indicate that adult females have a higher risk of developing resistance to available steroid therapies^4,5^ and are consequently more likely to suffer from uncontrolled severe asthma^6^. Essentially, heightened immune responses in females manifests into higher susceptibility to autoimmune, inflammatory and allergic disease, when compared to males^7–9^. To that end, sex-related differences in inflammatory processes in the lungs could drive sex-related disparities in the prevalence and severity of airway inflammation, which is pertinent to chronic respiratory disease such as asthma^1,10^. Although sex dimorphism is associated with the pathophysiology and response to therapy in asthma, sex as a biological variable is largely ignored in most preclinical studies using murine models. Therefore, in this study we characterized immune responses in the allergen house dust mite (HDM)-challenged model of acute airway inflammation, using two different strains of mice.

Murine models of allergen challenge with ovalbumin (OVA) or HDM are predominantly used for preclinical studies in asthma, but the majority of these studies employ only female mice^11^. Nonetheless, some evidence of sex dimorphism related to airway inflammation has been reported from murine models, primarily in studies using OVA sensitized and challenged mice with a T-helper (Th) 2-skewed immune response. For example, some indices of airway inflammation are more pronounced in OVA-challenged female mice compared to males, including elevated levels of IL-4, IL-5, IL-10, IL-13 and TGF-β in the bronchoalveolar lavage fluid (BALF) and enhanced eosinophilic inflammation^12–15^. Female mice also show higher OVA-specific IgE levels than male mice^12,16^. Although, HDM is the most prevalent natural allergen with approximately 85% of asthmatics sensitized to HDM globally^17^, sex-related differences in HDM-mediated immune response and airway inflammation have not been comprehensively characterized in murine models. Adult female BALB/c mice challenged with HDM result in bronchial inflammation, airway remodeling and epithelial damage similar to that seen in human asthma^18–22^. In this study, we focus predominantly on sex-related differences in airway inflammation using a murine model of HDM challenge for 2 weeks. The clinical relevance of HDM as an allergen favours its use in murine models for preclinical studies.

The genetic background of mice also impacts the severity of airway inflammation, influencing cytokine levels and the distribution of immune cell infiltration^23,24^. Thus, it is likely that the mouse strain will also influence sex-related differences in murine models of airway inflammation. Given the paucity of data, there is a need to identify sex-specific inflammatory responses and protein markers in response to HDM in different strains of mice. Therefore, the goal of this study was to integrate sex as a biological variable and identify specific sex-related differences in HDM-mediated inflammatory responses, in two common mouse strains. Here, we compare immune responses in female and male mice of two strains, BALB/c and C57BL/6NJ, with and without HDM-challenge. Specifically, adult mice were subjected to 2 weeks of repeated HDM challenge as this results in robust airway inflammation without significant airway remodelling^18^. We characterized sex-related differences in leukocyte and cytokine profiles in the lungs. The findings reported in this study provide objective endpoints and molecular markers of sex dimorphism in HDM-mediated acute airway inflammation, in two commonly used murine strains. This will be a valuable resource for future studies that use murine models of airway inflammation, applicable to preclinical studies such as in asthma research.

## Materials and methods

### HDM-challenged mouse model of airway inflammation

The mouse model used in this study was approved by The University of Manitoba Animal Research Ethics Board (protocol number AC11394 (B2018-038)). All experiments were performed in accordance with relevant guidelines and regulations, and were compliant with the ARRIVE guidelines for experimental design and reporting of data^25^. Briefly, 7–8 week old female and male mice, BALB/c and C57BL/6NJ, were purchased from Charles River Laboratories. Mice were housed in the central animal care facility at the University of Manitoba. The animal care facility staff randomly assigned mice to cages with a maximum of five mice per cage. The commonly used C57BL/6J strain has a mutation in *NLRP12* (Nod-like receptor pyrin domain containing 12) which impairs neutrophil recruitment, whereas the C57BL/6NJ mice sub-strain does not have this mutation^26^. Therefore, we used the sub-strain C57BL/6NJ mice in this study. Following an acclimatization period of 1 week, mice were challenged with intranasal (i.n) daily administrations of 35 μL of 0.7 mg/mL whole HDM protein extract in saline (Greer Laboratories, Lenoir, NC, USA), which is 24.5 μg of HDM extract per mouse. The HDM extract used in this study was with low endotoxin level (~ 800 EU/vial)^27^. HDM instillations were performed in the morning between 10 am and noon for five consecutive days per week followed by a 2 day rest, for 2 weeks as previously described^18,28^. Mice were monitored daily for activity levels and grooming. Mice were sacrificed 24 h after the last HDM challenge for sample collection based on our previous studies^18,28^.

### Cell differential assessment in BALF

Mice were anesthetized with intraperitoneal (i.p) administration of sodium pentobarbital (90 mg/kg), tracheostomized and lungs were washed twice with 1 mL of cold saline each time. BALF samples were centrifuged (150×*g* for 10 min at room temperature). The cell pellet was resuspended in 1 mL sterile saline, and 100 µL of the cell suspension was placed in a Cytospin slide for differential cell counting using a modified Wright–Giemsa stain (Hema 3^®^ Stat Pack, Fisher Scientific, Hampton, NH, USA). Slides were imaged using a Carl Zeiss Axio Lab A1 microscope (Carl Zeiss, Oberkochen, Germany). Cell differentials were counted in 5 image frames at 20 × magnification per slide, blinded by two different personnel.

### Preparation of lung tissue lysates

The left lobe of murine lung was flash frozen in liquid nitrogen and stored at − 80 °C until use. The flash frozen lung tissue was homogenised on ice in Protein Extraction Reagent T-Per (Thermo Fisher Scientific, Rockford, IL, USA) containing protease inhibitor cocktail (Cell Signalling, Davers, MA, USA) using the Cole-Parmer LabGEN 125 homogenizer (Cole-Parmer Canada, Quebec, Canada). Lung tissue homogenates were centrifuged at 10,000×*g* for 10 min at 4 °C. Total protein concentration was determined in the tissue lysates (supernatants) using Bicinchoninic acid (BCA) assay (Thermo Fisher Scientific). The lysates were aliquoted and stored at − 80 °C until use.

### Assessment of cytokines

BALF and lung tissue lysates were used to examine the concentrations of a panel of 29 cytokines (IFNγ, IL-10, IL-12p70, IL-1β, IL-2, IL-4, IL-5, IL-6, KC/GRO, TNFα, IL-16, IL-17A, IL-17C, IL-17E/IL-25, IL-17F, IL-21, IL-22, IL-23, IL-31, MIP3α, IL-15, IL-17A/F, IL-27p28/IL-30, IL-33, IL-9, IP-10, MCP1, MIP1α and MIP2) using the V-PLEX Mouse Cytokine Meso Scale Discovery (MSD) assay, according to the manufacturer's instructions (Meso Scale Discovery, Rockville, MD, USA). Any values less than the lower limit of detection were assigned half the value of the lowest detectable standard. Data was analyzed using the Discovery Workbench 4.0 software (Meso Scale Discovery). As the levels of IL-16 detected by MSD in lung tissue lysate were above the highest detectable standard, lung tissue lysates were diluted 1:100 and the abundance of IL-16 was determined independently using ELISA (R&D Systems, Minnesota, USA).

### Assessment of total and HDM-specific IgE antibodies

Serum concentration of total IgE was assessed by a mouse uncoated ELISA Kit (Thermo Fisher, Catalog # 88-50460-22) according to the manufacturer’s instructions, wherein the serum samples were diluted 1:50 for BALB/c and 1:25 for C57BL/6NJ. HDM-specific IgE level was assessed using indirect ELISA as previously described by us^18^. Briefly, Costar™ 96-well flat-well high-binding plates (Thermo Fisher Scientific) were coated with 100 μL per well of HDM extract (10 μg/mL) in PBS and incubated overnight at 4 °C. The plates were blocked with 3% BSA (w/v) in PBS (200 µL/well), and incubated overnight at 4 °C. Serum samples were precleared by overnight incubation with Protein G Sepharose beads in a 2:1 ratio (40 µL of serum with 20 µL of beads per sample) at 4 °C. Pre-cleared serum samples were used to detect HDM-specific IgE, using biotin-anti-mouse IgE (1:5000 dilution in PBS containing 1% (w/v) BSA) as the secondary detection antibody. Avidin-HRP and TMB substrate solution was used for the colorimetric reaction. Optical densities were read at 450 nm with reference at 570 nm using a BioTek Synergy 4 Microplate reader.

### Statistical analysis

Data reported in this study were disaggregated by sex, based on SAGER guidelines for sex- and gender-based analyses^29,30^. Two-way analysis of variance (ANOVA) with Tukey’s multiple comparisons test was used for testing the effect of HDM and biological sex for cell differential assessments in BALF. Two-way ANOVA with Tukey’s multiple comparisons test was also used for statistical analysis of serum levels of total and HDM-specific IgE. Mann–Whitney *U* test was used to compare total cells and cell differential between males and females, in HDM-challenged mice compared to allergen-naive (HDM/naïve). For cell types where the cell count in BALF was zero in naïve, a denominator of 1 was used to calculate the ratio of HDM/naïve. Mann–Whitney *U* test was also used for comparative assessment of cytokine levels. Statistical analyses were performed using GraphPad Prism (version 9.1; GraphPad Software). A value of *p* < 0.05 was considered to be statistically significant.

## Results

### Serum level of HDM-specific IgE is significantly higher in female mice compared to males

**Figure 1:**
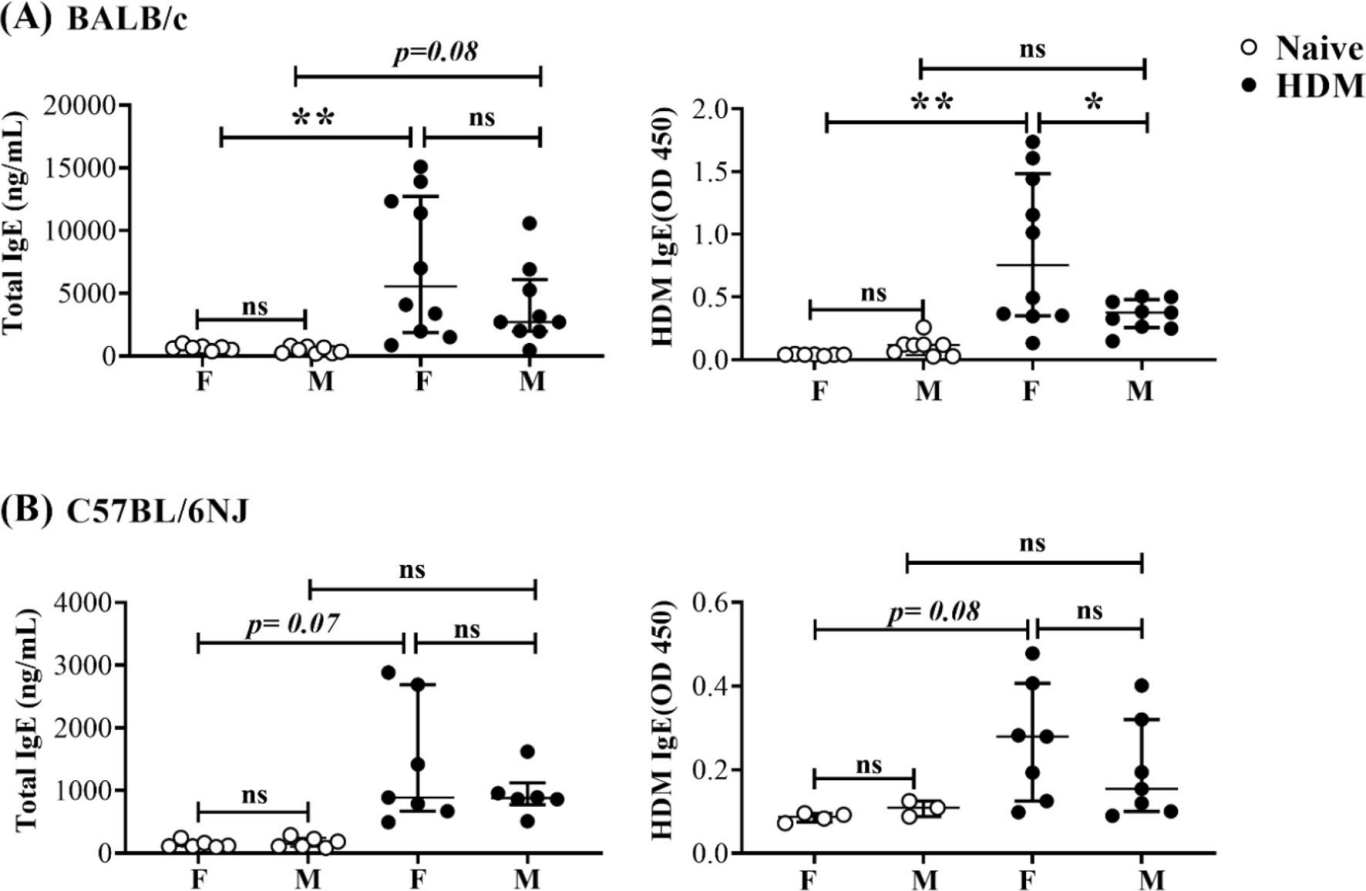
Total and HDM-specific IgE antibodies in serum. Mice were challenged (intranasal) with 35 μL of 0.7 mg/mL whole HDM protein extract in saline (i.n) per mouse, once daily for five consecutive days with a 2 day rest in between, for 2 weeks. Blood was collected from (**A**) BALB/c and (**B**) C57BL/6NJ mice, 24 h after the last HDM challenge and serum was used for assessment of total IgE and HDM-specific IgE antibodies by ELISA. Each dot represents an individual mouse. Female mice = F, and male mice = M. Statistical significance was determined using Two-way ANOVA with Tukey’s multiple comparisons test. (*p ≤ 0.05, **p ≤ 0.01, ns = non-significant).

IgE antibodies play a pivotal role in propagating airway inflammation following allergen exposure^31^. We have previously shown that HDM-specific IgE antibodies are enhanced in adult female BALB/c mice following repeated instillation of HDM for 2 weeks^18^. Thus, we assessed serum levels of total and HDM-specific IgE. There were no significant differences in the serum levels of total IgE and HDM-specific IgE between naïve female and male mice, in both strains (Fig. 1). HDM-challenge resulted in the increase of total IgE serum levels in BALB/c mice, which was statistically significant in female mice (*p* < 0.01) and showed a trend of increase (*p* = 0.08) in males (Fig. 1A). HDM-specific IgE levels significantly increased in serum obtained from only female but not male BALB/c mice, following HDM challenge (Fig. 1A). There was a trend in the increase of total IgE (*p* = 0.07) and HDM-specific IgE (*p* = 0.08) in female C57BL/6NJ mice, in response to HDM (Fig. 1B). Whereas the circulating levels of total or HDM-specific IgE did not increase in male C57BL/6NJ mice (Fig. 1B). These results indicated that HDM challenge results in an increase of HDM-specific serum IgE selectively in female mice, after 2 weeks. However, lung function assessments did not show any significant sex-related differences following HDM challenge in this study (Supplementary Fig. 1). It is likely that a longer duration of HDM challenge of at least 4 weeks may be required to comprehensively detail sex disparities in HDM-mediated systemic responses such as IgE antibody production as well as changes in lung physiology^22^. Therefore, we further focused on sex-related differences in local mucosal immunological responses, and comprehensively examined molecular endpoints of airway inflammation such as leukocyte differentials and cytokine profiles in the lungs.

### Sex-related differences in leukocyte accumulation in the lungs in response to inhaled HDM

We have previously shown that repeated instillation of HDM for 2 weeks results in a significant increase in specific leukocytes such as eosinophils and neutrophils, which are known to promote airway inflammation, in the BALF of female BALB/c mice^18,28^. Based on our previous studies, we examined total cell counts and leukocyte differentials in BALF samples obtained from female and male mice, 24 h after the last HDM challenge. In naïve mice of both strains (BALB/c and C57BL/6NJ) the primary cell type in BALF at baseline was macrophages (~ 90% of total cells), with no significant sex-related differences (Supplementary Fig. 2). There were no statistically significant differences in total cell accumulation, or for any of the leukocytes examined, between female and male naïve mice of both strains (Supplementary Fig. 3). Following repeated HDM instillation for 2 weeks, eosinophils were the major cell type in the BALF (~ 55% of total cells) of both strains of mice (Supplementary Fig. 2). To account for any variability in cell counts at basal level in naïve mice (Supplementary Fig. 3), we assessed the ratio of cell counts in each HDM-challenged mouse compared to the average count from the naïve group (HDM/naïve), for total cells and each cell type examined in the BALF.

**Figure 2:**
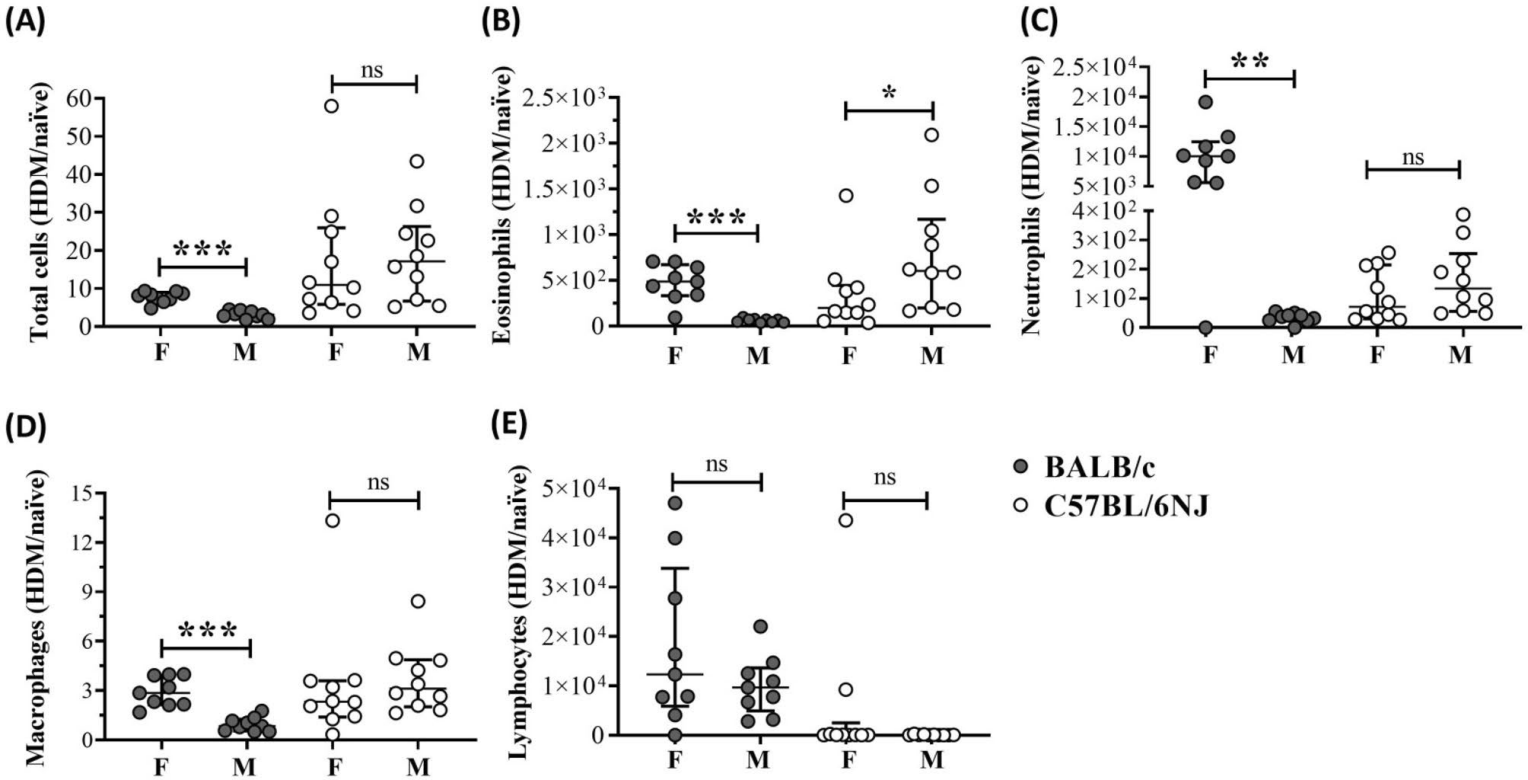
Leukocyte differentials in bronchoalveolar lavage fluid (BALF). BALB/c mice (n = 9 each, of females and males, per group) and C57BL/6NJ (n = 10 each, of females (F) and males (M), per group) were challenged (i.n) with 35 μL of 0.7 mg/mL whole HDM protein extract in saline, per mouse, once daily for five consecutive days with a 2 day rest in between, for 2 weeks. BALF was collected 24 h after the last HDM challenge. (**A**) Total cells, (**B**) eosinophils, (**C**) neutrophils, (**D**) macrophages and (**E**) lymphocytes were assessed in BALF using a modified Wright–Giemsa stain. Graphs represent ratio of cell counts in BALF obtained from each HDM-challenged mouse compared to the mean value from the naïve group (HDM/naïve) for each strain. Mann–Whitney *U* test was used to compare the ratio (HDM/naïve) of cell counts between females and males (*p = 0.05, **p = 0.01, ns = non-significant).

In female BALB/c mice, total cell accumulation was higher by ~ 3-fold (*p* < 0.0001), eosinophils by ~ 8-fold (*p* < 0.0001) and macrophages by ~ 3-fold (*p* < 0.0001) compared to males, in response to HDM (Fig. 2 and Supplementary Table I). Neutrophil accumulation in the BALF of female BALB/c mice were > 250-fold higher (*p* < 0.003) compared to males, in response to HDM (Fig. 2C and Supplementary Table I). These effects were not observed in C57BL/6NJ mice. In contrast to BALB/c, HDM-driven eosinophil accumulation in the BALF of male C57BL/6NJ mice was ~ 2-fold higher compared to females (Fig. 2B and Supplementary Table I). These results demonstrated that overall composition of leukocytes in the lungs, as well as specific sex-related differences in response to HDM, are dependent on the strain of the mice.

### Sex-related differences in lung cytokine profile in naïve mice

To define sex-related differences in cytokine profiles in the lung, we measured the abundance of 29 cytokines and chemokines in BALF and lung tissue lysates using the multiplex MSD platform. Out of the 29 cytokines measured, 18 were detected in the BALF and all 29 were detected in the lung tissue lysates. There were distinct sex-related differences in the abundance of specific cytokines at baseline levels in the lungs of naïve mice of both strains.

**Figure 3:**
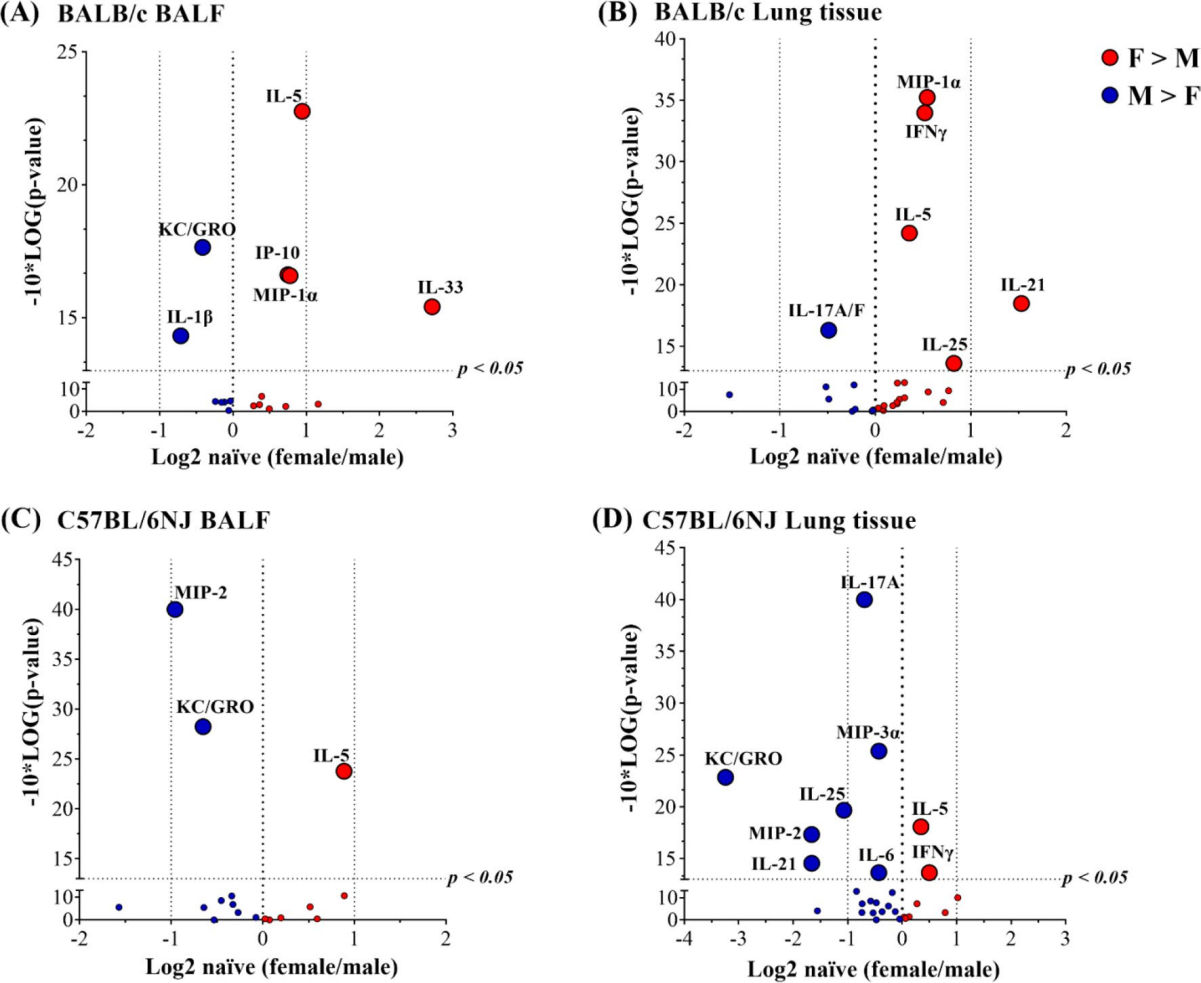
Sex-related differences in cytokine abundance in the lung of naïve BALB/c and C57BL/6NJ mice. A panel of 29 cytokines were measured by multiplex Meso Scale Discovery (MSD) platform in (**A**) BALF and (**B**) lung tissue lysates obtained from naïve BALB/c mice (n = 9 each, female and male), and (**C**) BALF and (**D**) lung tissue lysates obtained from naïve C57BL/6NJ mice (n = 10 each, female (F) and male (M) mice). Log2 values of average concentrations (pg/mL) of each cytokine were used for the volcano plots to examine the differences between female and male mice. Cytokine abundances that were significantly higher in females compared to males have positive values (shown in red), and those that were significantly higher in males compared to females have negative values (shown in blue), as represented on the x-axis of the volcano plots. Dotted lines represent a fold change of 2 (x-axis) and *p* < 0.05 (y-axis).

BALF of naïve female BALB/c mice had significantly higher levels (~ 2-fold) of IL-5, IP-10 and MIP-1α, and 4-fold higher IL-33 abundance, compared to males (Fig. 3A). The lung tissue lysates of naïve female BALB/c mice had significantly higher levels (< 2-fold) of IFNγ, MIP-1α, IL-5 and IL-25, whereas IL-21 was > 2-fold higher in females, compared to males (Fig. 3B). Naïve male BALB/c mice had significantly higher levels (< 2-fold) of IL-1β and KC/GROα in the BALF (Fig. 3A), and IL-17A/F (< 2-fold) in the lung tissue lysates (Fig. 3B), compared to females.

Naïve female C57BL/6NJ mice showed significantly higher level of IL-5 (~ 2-fold in the BALF and < 2-fold in lung tissue lysates) compared to males (Fig. 3C,D respectively). IFNγ was also significantly higher in the lung tissue lysates of naïve female C57BL/6NJ mice (Fig. 3D). Naïve male C57BL/6NJ mice had significantly higher levels (< 2-fold) of MIP-2 and KC in the BALF, compared to females (Fig. 3C). The lung tissue lysate of naïve C57BL/6NJ male mice demonstrated a robust sex bias, with the abundance of KC, MIP-2, IL-21 and IL-25 higher (> 2-fold), and IL-17A and MIP-3α (< 2-fold higher), compared to females (Fig. 3D).

Comparative analysis of these cytokine profiles (Fig. 3) revealed that IL-5 was significantly higher at baseline levels in the lungs (BALF and lung tissue lysates) of females compared to males, in both BALB/c and C57BL/6NJ mice (Fig. 3). With the exception of lung tissue lysates from BALB/c mice, KC/GROα was higher in naïve males compared to females, in both strains of mice (Fig. 3). Similarly, IL-17 was significantly higher in lung tissue lysates of naïve males compared to female, in both strains of mice (Fig. 3B,D), albeit IL-17A/F was noted in BALB/c and IL-17A in C57BL/6NJ mice. These results clearly demonstrated sex- and strain-related differences in the cytokine profile of the lungs at baseline levels in naïve mice. Based on these results we corrected for baseline values to assess HDM-driven change in cytokine abundance as follows.

### Sex-related differences in HDM-mediated increase of secreted cytokines in the BALF

**Figure 4:**
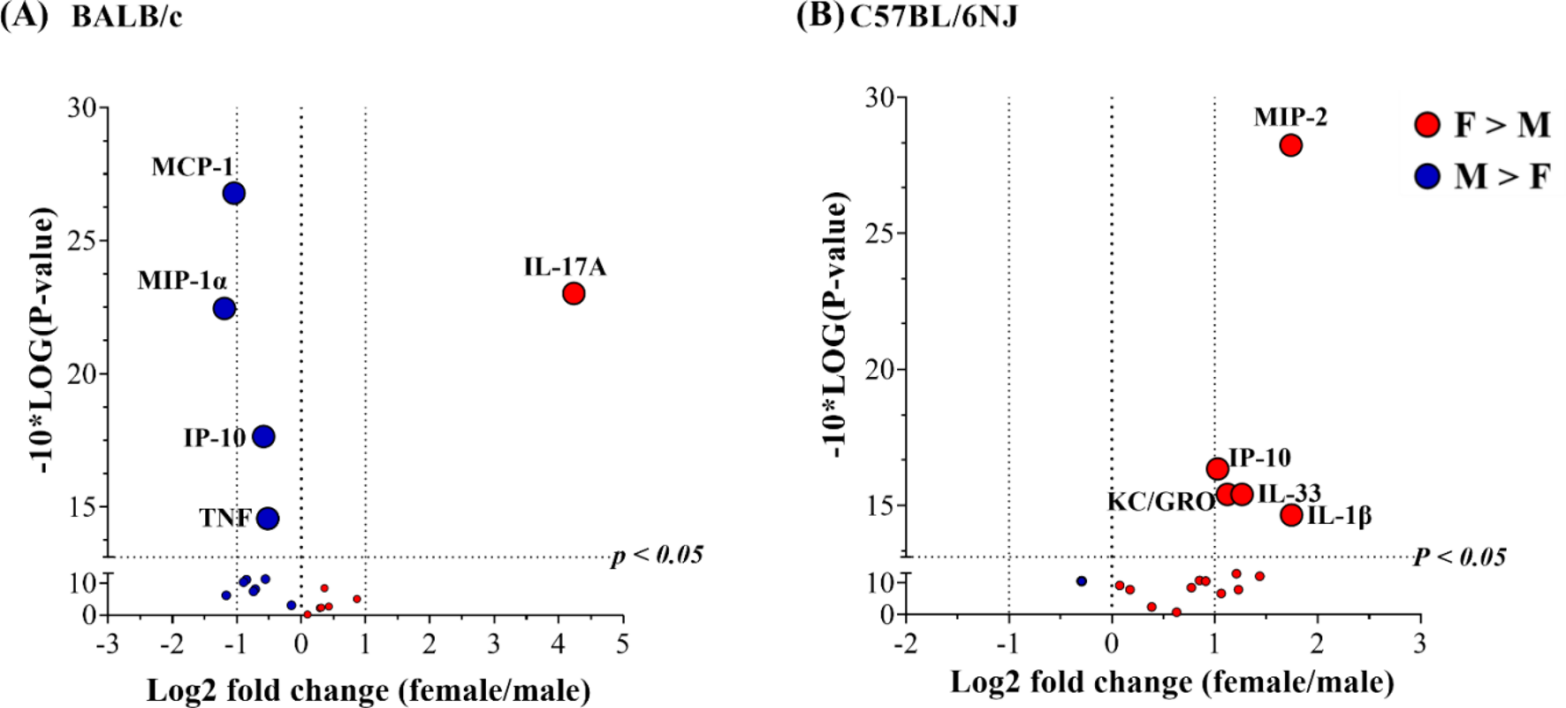
Sex-related differences in HDM-mediated secreted cytokines in BALF. (**A**) BALB/c mice (n = 9 each, of females (F) and males (M), per group) and (**B**) C57BL/6NJ mice (n = 10 each, of females and males, per group) were challenged (i.n) with 35 μL of 0.7 mg/mL whole HDM protein extract in saline per mouse, once daily for five consecutive days with a 2 day rest in between for 2 weeks. A panel of 29 chemokines and cytokines were measured in BALF collected 24 h after the last HDM challenge, by multiplex Meso Scale Discovery (MSD) platform. The ratio of concentration of each cytokine in the BALF of each HDM-challenged mouse compared to the mean concentration obtained from the group of naïve mice represents the HDM-driven fold change (HDM/naïve). Log2 values of average HDM/naïve fold change obtained from female and male BALF were used for the volcano plots. Positive values on the x-axis of the volcano plots (shown in red) are cytokines that are significantly higher in females compared to males, and negative values (shown in blue) are those that are significantly higher in males. Dotted lines represent a fold change of 2 (x-axis) and *p* < 0.05 (y-axis).

To determine HDM-driven change for each cytokine measured, we assessed the ratio of concentration in the BALF obtained from each HDM-challenged mouse compared to the mean concentration in BALF from the group of naïve mice i.e. HDM/naïve (Supplementary Table II). To determine sex-related differences, we compared the mean HDM/naïve values for each cytokine between female and male mice, in both strains of mice independently (Fig. 4 and Supplementary Table II). Of the 29 cytokines examined, 16 cytokines were increased by > 2-fold in the BALF of female and male mice of both strains, in response to HDM (Supplementary Table II), with specific sex-related differences as follows.

Strikingly, the HDM-driven increase in IL-17A was > 150-fold (*p* < 0.01) in females and only 8-fold in male BALB/c mice, compared to naïve mice (Supplementary Table II). IL-17A was the only cytokine that was significantly higher (by ~ 16-fold) in the BALF of female BALB/c mice compared to males following HDM challenge (Fig. 4A). This result indicates that HDM drives a T-helper (Th)-17-skewed response in the BALF of female BALB/c mice compared to males. In contrast, male BALB/c mice had significantly higher levels (by ~ 2-fold) of TNF, IP-10, MCP-1 and MIP-1α in the BALF compared to females (Fig. 4A and Supplementary Table II), indicating a Th1-biased response in the secreted protein profile.

In contrast to BALB/c, the HDM-driven secreted cytokine profile in the BALF of C57BL/6NJ mice demonstrated a clear female-bias with significantly higher levels of IL-1β, KC/GRO, IL-33, IP-10 and MIP-2 (between 2 and 4-fold) compared to males (Fig. 4B and Supplementary Table II). None of the cytokines examined were significantly higher in the BALF of male C57BL/6NJ mice compared to females (Fig. 4B). These results indicated that HDM results in significantly higher levels of specific Th1-bias cytokines in the BALF of female C57BL/6NJ mice compared to males.

### Sex-related differences in HDM-mediated increase of cytokine abundance in lung tissues

**Figure 5:**
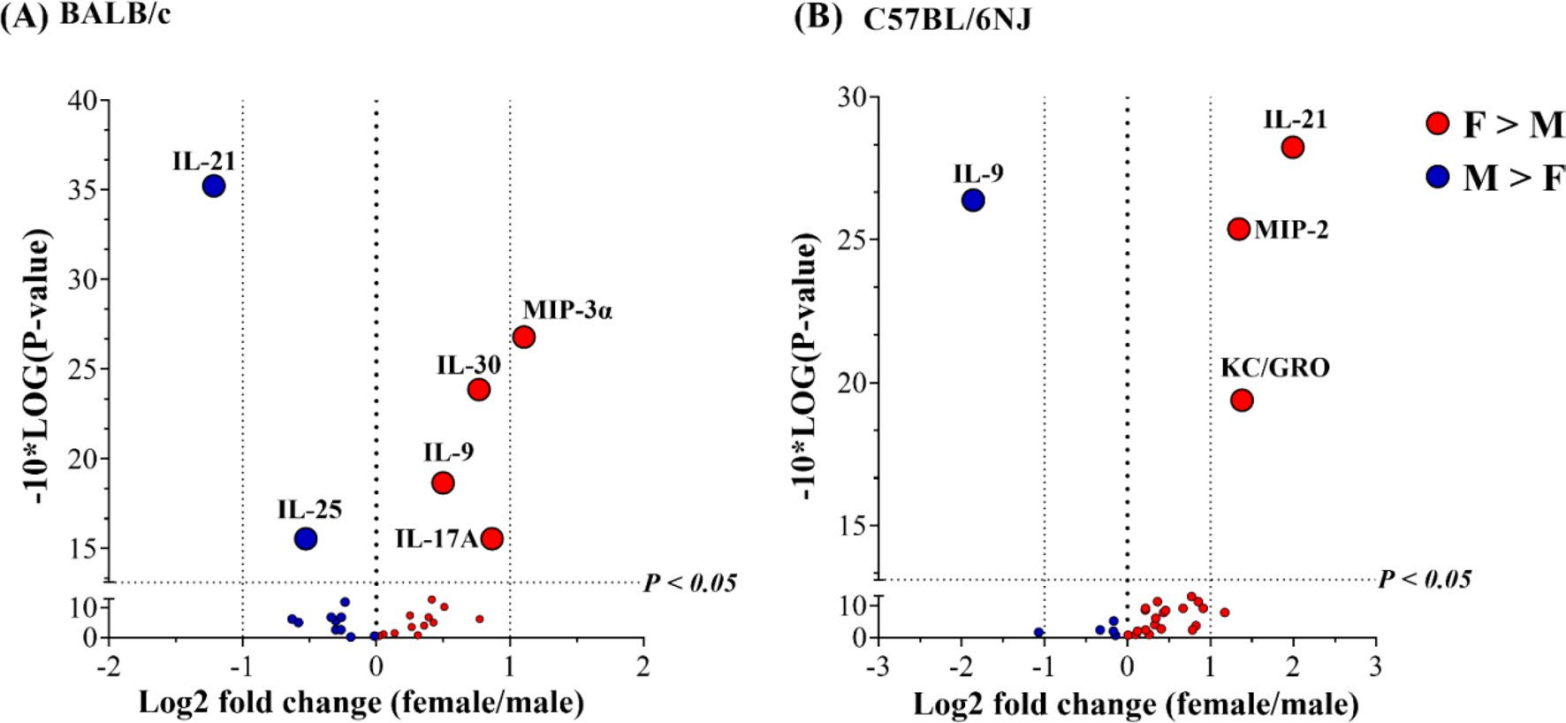
Sex-related differences in HDM-mediated increase in the abundance of cytokines in lung tissue lysates. (**A**) BALB/c mice (n = 9 each, of females (F) and males (M), per group) and (**B**) C57BL/6NJ mice (n = 10 each, of females and males, per group) were challenged (i.n) with 35 μL of 0.7 mg/mL whole HDM protein extract in saline per mouse, once daily for five consecutive days with a 2 day rest in between for 2 weeks. A panel of 29 chemokines and cytokines were measured in the lung tissue lysates collected 24 h after the last HDM challenge, by the multiplex Meso Scale Discovery (MSD) platform. The ratio of the abundance of each cytokine in the lung tissue lysates from each HDM-challenged mouse compared to the mean abundance obtained from the group of naïve mice represents the HDM-driven fold change (HDM/naïve). Log2 values of average HDM/naïve fold change obtained from female and male BALF were used for the volcano plots. Positive values on the x-axis of the volcano plots (shown in red) are cytokines that are significantly higher in females compared to males, and negative values (shown in blue) are those that are significantly higher in males. Dotted lines represent a fold change of 2 (x-axis) and *p* < 0.05 (y-axis).

Similar to that described above for BALF, we assessed the ratio of abundance in the lung tissue lysates obtained from each HDM-challenged mouse compared to the mean abundance in lung tissue lysates from the group of naïve mice i.e. HDM/naïve (Supplementary Table III). To determine sex-related differences, we compared the mean HDM/naïve values for each cytokine between female and male mice in both strains of mice independently (Fig. 5 and Supplementary Table III). The abundance of 19 cytokines in BALB/c and 23 cytokines in C57BL/6NJ were significantly increased in the lung tissue lysates, in response to HDM challenge compared to naïve mice (Supplementary Table III).

HDM-driven increase in the abundance of IL-17A, MIP-3α, IL-30 and IL-9 were significantly higher (between 1.5 and 2-fold) in the lung tissue lysates of female BALB/c mice compared to males (Fig. 5A). Male BALB/c mice had significantly higher abundance of IL-21 (> 2-fold) and IL-25 (< 2-fold) in lung tissue lysates compared to females (Fig. 5A).

In contrast to BALB/c mice, female C57BL/6NJ mice did not demonstrate an IL-17-skewed response in response to HDM. Instead, female C57BL/6NJ mice showed significantly higher (> 2-fold) abundance of KC/GRO, MIP-2 and IL-21 in the lung tissue lysates compared to males, in response to HDM (Fig. 5B). IL-9 was the only cytokine that was significantly higher (~ 4-fold) in the lung tissue lysates of male C57BL/6NJ mice compared to females, in response to HDM (Fig. 5B).

## Discussion

**Figure 6:**
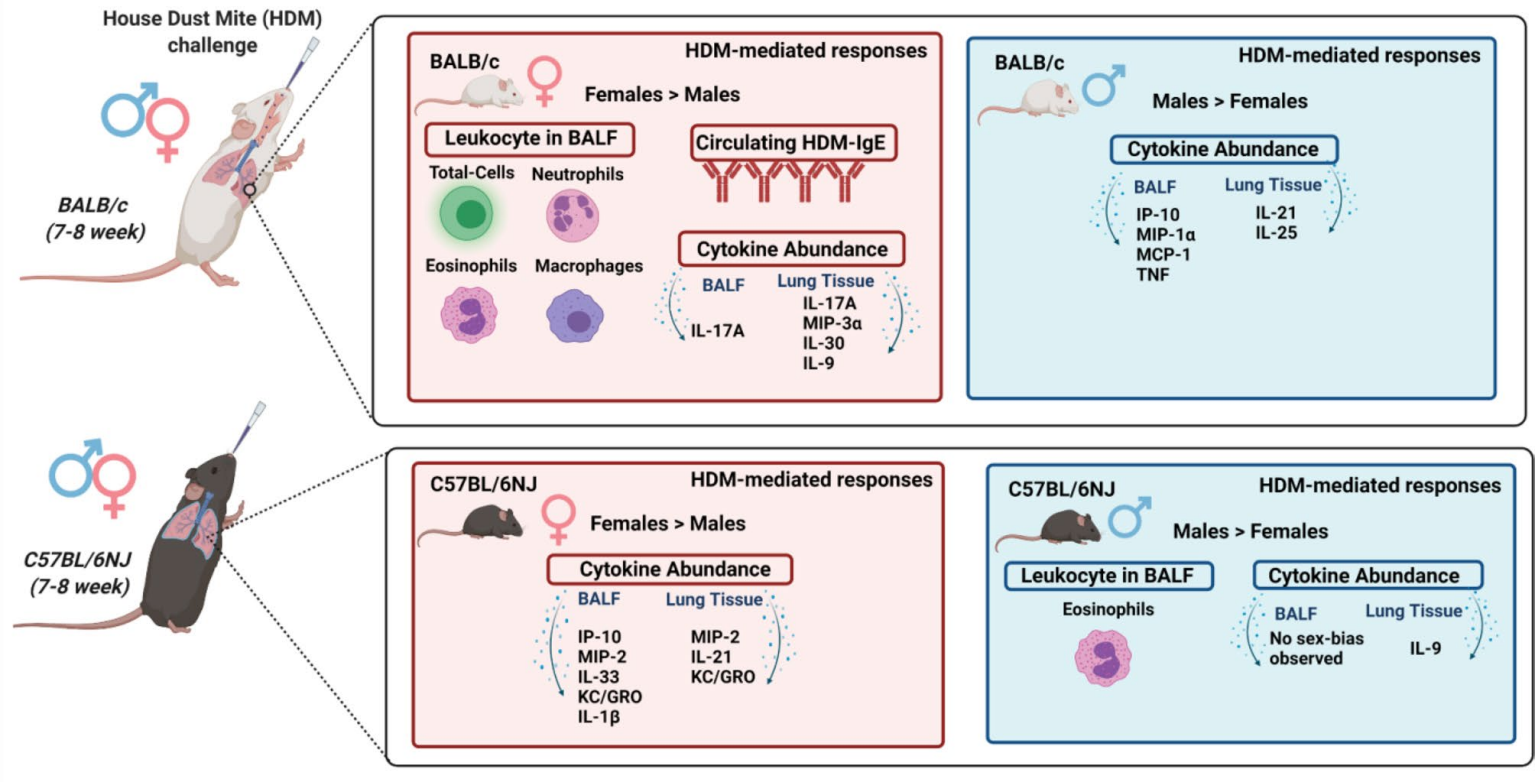
Sex dimorphism in HDM-mediated leukocyte accumulation and cytokine profile in the lungs is strain dependent. This figure summarizes sex-related differences in leukocyte accumulation and the increase in specific cytokines in BALB/c and C57BL/6NJ mice. HDM-mediated increase in the accumulation of total cells, neutrophils, eosinophils and macrophages are higher in female BALB/c mice compared to males. Whereas, male C57BL/6NJ mice have higher accumulation of eosinophils compared to females in response to HDM. Cytokine profile assessments indicate that HDM-driven increase in IL-17 and MIP-3α (a chemoattractant of Th-17 cells) is significantly higher in female BALB/c mice compared to males, suggesting a Th17-skewed response. Whereas HDM-driven cytokine profiles in female C57BL/6NJ mice indicate a Th1-skewed response compared to males. In contrast, HDM-mediated cytokine profiles in male mice of both strains show higher levels of Th2-associated cytokines compared to females (created using https://BioRender.com).

Sex is an important biological variable in the development, severity and response to therapy in asthma^1–3^. Thus, it is important to define sex-related differences in response to inhaled allergens in different animal models that are routinely used in preclinical studies for asthma. In this study, we characterized sex dimorphism in the allergen HDM-mediated immune responses in a murine model predominantly resulting in airway inflammation, in two different strains of adult mice (BALB/c and C57BL/6NJ). Allergen-mediated pathophysiology of disease such as allergic asthma is heterogeneous and cannot be fully captured by a single animal model. Thus, interpretation of findings in animal model studies need to be in the context of the allergen used, the route and duration of allergen challenge, and the time point selected for endpoint assessments. The murine model used in this study with repeated HDM instillations for 2 weeks results in airway inflammation preceding fibrosis^18^. Therefore, the sex-related differences reported in this study are primarily in the context of airway inflammation. Here, we demonstrate sex dimorphism in HDM-driven leukocyte accumulation and cytokine profile in the lungs with notable strain-dependent differences (summarized in Fig. 6). We also show that there are sex-related differences in specific cytokines at the basal level in naïve BALB/c and C57BL/6NJ mice, which needs to be taken into consideration for interpretation of data in mouse models. Overall, the findings of this study provide objective sex- and strain-specific differences in specific cytokines and leukocytes, which will be a valuable resource for research in allergic airway inflammation and applicable to preclinical studies in asthma using various mouse models.

To our knowledge, this is the first study to provide a comprehensive comparative assessment of cytokine profiles that defines sex-related differences in HDM-mediated airway inflammation, in two different mouse strains. Previous studies detailing sex-related differences were primarily focused on Th2-skewed cytokines^12–16,32,33^. Here, we detail the abundance of cytokines in BALF and lung tissue lysates using an unbiased approach. Firstly, our results demonstrate that specific inflammatory cytokines show a sex-bias at baseline levels in naïve mice. Notably, IL-5 is significantly higher in the BALF and lung tissue lysates of naïve females compared to males, in both strains of mice examined. IL-5 plays a critical role in the induction and amplification of eosinophilic inflammation in asthma^34^. Transcriptomics of innate lymphoid cells (ILC)-2, producers of IL-5, reveals that lung ILC2s are more metabolically active in naïve post-pubertal female mice, and are more prone to be activated by inflammatory challenge compared to males^35^. As ILC2s play a role in HDM-mediated airway inflammation in mice^36^, it is possible that higher basal levels of IL-5 indicate that females are more primed to respond to inhaled allergen challenge compared to males. Cellular composition of BALF provides insight into the ongoing inflammatory state in the lungs^37^. Indeed, our findings show that HDM-driven accumulation of inflammatory leukocytes in the lungs is significantly higher in females compared to male mice, in particular in BALB/c mice, suggesting that allergen-driven airway inflammation in females is more robust compared to males. This is also aligned with our results demonstrating a higher systemic antibody response to HDM in females compared to males in both murine strains. In contrast to naïve female mice, we demonstrate that IL-17 (IL-17A and IL-17A/F), known to promote Th17-high neutrophilic inflammation^38,39^, is higher in the lungs of naïve male mice in both strains. However, the HDM-driven increase in IL-17 was not higher in male mice compared to females in both strains of mice. These findings clearly demonstrate sex-related variance in basal levels of specific cytokines in naïve mice of both strains. Moreover, our findings indicate that sex-related variances in the basal levels of cytokines do not lead to similar differences in response to inhaled allergen. Thus, our results provide a strong rationale for considering cytokine abundance at basal levels in naïve mice while defining sex-related differences in cytokine profiles of the lungs in murine studies.

In this study, we show that HDM drives a mixed eosinophil-neutrophil cellular phenotype along with significantly higher IL-17A abundance in the lungs of female BALB/c mice compared to males. IL-17A was the only cytokine with a clear female-bias in BALB/c mice, being significantly higher in the BALF and lung tissue lysates of females compared to males, in response to HDM. IL-17A is associated with amplifying the severity of allergen-induced airway inflammation, particularly neutrophilia^38,39^, and contributes to glucocorticoid insensitivity in human bronchial epithelial cells^40^. Moreover, adult female asthma patients have a higher prevalence of steroid-unresponsive asthma compared to males^41–44^. Our results corroborate these observations, wherein we show that HDM drives a Th17-skewed immune response with significantly higher levels of IL-17 and neutrophil accumulation in the BALF of female BALB/c mice compared to males. Some previous studies have also reported higher HDM-induced lung IL-17A expression in female BALB/c mice^33,45^. In addition, we show that the HDM-mediated increase in MIP-3α is significantly higher in the lung tissue lysates of female BALB/c mice compared to males. MIP-3α (CCL20), produced by macrophages and epithelial cells, is a chemoattractant of Th17 cells and neutrophils, and is found to be elevated in the sputum of asthmatics^42,43^. MIP-3α is also associated with glucocorticoid-insensitive inflammation in asthma^46^. The molecular mechanisms that underpin the role of IL-17 and MIP-3α in asthma severity are not completely defined. Our results suggest that inhaled HDM exposure results in higher levels of MIP-3α, which could drive the enhancement of IL-17 and neutrophil accumulation in the lungs. Thus, MIP-3α may be a critical factor facilitating a higher prevalence of severe asthma in adult females which needs to be further elucidated.

Furthermore, results of this study clearly demonstrate that HDM-mediated changes in the lung cytokine profile are murine strain-dependent. For example, in contrast to BALB/c mice, HDM-driven changes in the lung cytokine profile primarily show a Th1-skewed response in C57BL/6NJ female mice with higher levels of MIP-2, IP-10, IL-1β and KC /GROα compared to males. Moreover, cytokines known to promote Th2-effector functions in airway inflammation such as IL-33 (in BALF) and IL-21 (in lung tissue)^47,48^ are also higher in female C57BL/6NJ mice compared to males, in response to HDM. These results indicate that female C57BL/6NJ mice elicit a mixed Th1/Th2-skewed response following HDM challenge, which is distinctly different from that in BALB/c mice. However, our findings are different from a recent study by Weiss et al., which showed that HDM predominantly drives a Th2-biased response with elevated levels of IL-4 and IL-5 in females compared to males using an allergen recall model in C57BL/6J mice^49^. These discrepancies are most likely due to the differences in the dose and duration of HDM challenge, and the substrain of mice used. This emphasizes the importance of taking the dose and duration of the allergen challenge into consideration while interpreting data related to sex dimorphism in mouse models. In addition, the study by Weiss et al. housed mice in pathogen-free facility^49^, which was not the case in this study, thus highlighting the influence of microbiome in altering the profile of inflammatory response in the lungs, which warrants further investigation.

Interestingly, lung tissue lysates obtained from male mice of both strains show significantly higher level of cytokines associated with Th2-mediated airway inflammation compared to females, in response to HDM. For example, HDM-mediated increase in IL-21 and IL-25 were higher in the lung tissue lysates of male BALB/c mice compared to females. IL-21 plays a critical role in Th2 cell survival and polarization^48^, whereas IL-25 induces and augments Th2-inflammatory responses^50^. Similarly, IL-9, which promotes a Th2-skewed immune response by inducing the production of IL-13, and eosinophilic airway inflammation^51,52^, was higher in the lung tissue lysates of male C57BL/6NJ mice compared to females, in response to HDM. Taken together, these results suggest that HDM-drives a higher Th2-skewed cytokine profile in the lungs of male mice compared to females, albeit with specific differences between the two mice strains used in this study. Our results are corroborated by previous studies demonstrating murine strain-dependent differences in airway inflammation in OVA-sensitized models^24,53,54^. Similarly, HDM-mediated airway inflammation and lung pathophysiology was also shown to be influenced by the genetic background of the mice^55^. Note that we do not report significant sex-related differences in lung function or airway hyperresponsiveness in this study. It is likely that a chronic model of HDM challenge or an allergen recall model, with allergen challenge for at least 4 weeks^22^, is required to expansively detail sex disparities in lung physiology and fibrosis related to HDM-mediated pathophysiology in future studies. Nevertheless, our results point to an important caveat in the interpretation of cytokine data from murine models, highlighting that findings related to sex disparity in allergen-mediated airway inflammation need to take the strain of mouse into consideration while translating data from preclinical studies.

Sex dimorphism in lung cytokine profiles that is reported here indicates that HDM primarily drives a Th17-skewed response in female BALB/c mice, and that female C57BL/6NJ mice mount a higher Th1/Th2 mixed response compared to males (Fig. 6). In contrast, male mice of both strains show higher levels of Th2-associated cytokines in the lungs in response to HDM (Fig. 6). There is a robust link between cytokine response and asthma endotypes which are extremely heterogenous, ranging from a Th2-driven to a mixed Th1/Th17-driven disease^56–60^. Mouse models associated with various endotype-related immunophenotypes do not directly capture the heterogeneity of asthma patients^61^. To that end, this study provides two different strain-dependent systems which may be used to delineate mechanisms that underpin sex dimorphism in disparate airway inflammatory phenotypes in murine models. A caveat of this study is that the HDM challenge model used here is predominantly associated with acute airway inflammation. Thus, the findings of this study are applicable to preclinical studies using murine models of diseases characterized by acute airway inflammation such as in allergic asthma. As mentioned above, allergen-mediated airway inflammation results in heterogeneous immunophenotypes. Therefore, murine models of chronic HDM challenge or allergen recall protocols may results in different inflammatory profiles, wherein sex disparities in cytokine responses differ from the results highlighted in this study. Also, a chronic model of HDM challenge will be required to delineate sex dimorphism in airway remodeling and fibrosis associated with asthma. Nevertheless, findings in this study clearly highlight the need for integrating sex as biological variable in animal model studies using HDM challenge for preclinical asthma research.

Another important caveat in this study is the use of post-pubertal mice. Sex bias in asthma prevalence and severity is associated with age, with a sex shift around puberty wherein the prevalence and severity of the disease is higher in adult females compared to males^62^. Thus, the results reported in this study will most likely be relevant to sex-related differences in adults. It is possible that the sex dimorphism in HDM-mediated airway inflammation is associated with differences in sex steroids. Although sex steroids such as oestrogen have been associated with pro-inflammatory responses in asthma^63^, this association is confounding. Oestrogen can induce immune activation to promote inflammation, and in contrast also exert overall anti-inflammatory effects^64,65^. Thus, the association of sex steroids with HDM-mediated airway inflammation needs to be further delineated (which is beyond the scope of this study).

In summary, the findings reported in this study highlight the importance of considering sex as a biological variable in the experimental design and interpretation of data in murine models of allergen-challenged airway inflammation, such as those typically used for preclinical studies in asthma. Moreover, this study demonstrates that sex dimorphism in airway inflammation is also strain-dependent in mouse models. The sex-related differences defined in this study provide a HDM-induced biosignature, consisting of a panel of unique and overlapping quantifiable cytokines, in both BALB/c and C57BL/6NJ mice, in a model particularly associated with acute airway inflammation. The findings reported in this study provide the foundation for future studies to delineate mechanisms that underpin sex-related differences in allergen-mediated airway inflammation, and to examine sex dimorphism in response to new interventions, using different mouse models of HDM challenge. Overall, the results of this study can serve as an important resource that is directly applicable to translational research using mouse models of allergen-induced airway inflammation in preclinical studies for asthma.

## Supplementary Information

Supplementary Information.

## Data Availability

All data generated and analysed during this study are included in this published article and its supplementary information files.
