# Supplementary material for: Characterization of sex-related differences in allergen house dust mite-challenged airway inflammation, in two different strains of mice
Source: Sci Rep. 2022 Dec 2;12:20837. doi: 10.1038/s41598-022-25327-7 (PMC9718733; doi:10.1038/s41598-022-25327-7)
Supplement: Supplementary file 1 — Supplementary Information. [file 41598_2022_25327_MOESM1_ESM.docx]

**SUPPLEMENTAL INFORMATION**

**Characterization of sex-related differences in allergen house dust mite-challenged airway inflammation, in two different strains of mice**

Dina H. D. Mostafa^1,2^, Mahadevappa Hemshekhar^2^, Hadeesha Piyadasa^1,2,3^, Anthony Altieri^1,2^, Andrew J. Halayko^4,5^, Christopher D. Pascoe^4,5,#^ and Neeloffer Mookherjee^1,2,5,#,*^

^1^Department of Immunology, University of Manitoba, Winnipeg, Manitoba, Canada.

^2^Manitoba Centre for Proteomics and Systems Biology, Department of Internal Medicine, University of Manitoba, Winnipeg, Manitoba, Canada.

^3^Department of Pathology, School of Medicine, Stanford University, Palo Alto, California 94304, United States.

^4^Department of Physiology and Pathophysiology, University of Manitoba, Winnipeg, Manitoba, Canada.

^5^Biology of Breathing Group, The Children’s Hospital Research Institute of Manitoba, Winnipeg, Manitoba, Canada.

^#^Co-Senior authors

^*^Corresponding author:

Dr. Neeloffer Mookherjee,

799 JBRC, 715 McDermot Avenue, Winnipeg, MB R3E 3P4, Canada.

Tel +1-204-272-3115. Fax +1-204-480-1362. Email: neeloffer.mookherjee@umanitoba.ca

**Short title:** Biological sex and airway inflammation

**Key words:** Allergen, Asthma, Lungs, Sex, Cytokines, House dust mite, Inflammation, Mouse.

**(I) FIGURES**

**Supplementary Figure 1: Lung function assessment in BALB/c and C57BL/6NJ mice. (A)** BALB/c mice and **(B)** C57BL/6NJ mice (n=5 each, female and male, per group) were challenged (i.n) with 35 μL of 0.7 mg/mL whole HDM protein extract in saline per mouse, once daily for 5 consecutive days with two day rest in between for 2 weeks. Mice were anaesthetised and tracheotomized 24 hour after the last HDM challenge, and lung mechanics was assessed using a flexiVent™ small animal ventilator (SCIREQ Inc, Montreal, QC, Canada). Mice were subjected to nebulized saline for baseline measurement followed by an increasing dose of nebulized methacholine (3–50 mg/ml). Lung mechanics were assessed using high frequency forced oscillation technique with positive end-expiratory pressure of 3 cmH2O in order to measure total resistance (Rrs) for resistance in the lung, Newtonian resistance (Rn) for central airway resistance, tissue damping (G) to monitor alveolar tissue restriction, and tissue elastance (H) to determine the stiffness of tissue. Changes in Rrs, Rn, G and H were monitored in response to nebulized saline and increasing concentrations of nebulized methacholine using the Quick Prime-3 and Snapshot perturbations. Data was collected using flexiWare Software and transferred to Microsoft Excel and GraphPad Prism software for analysis.


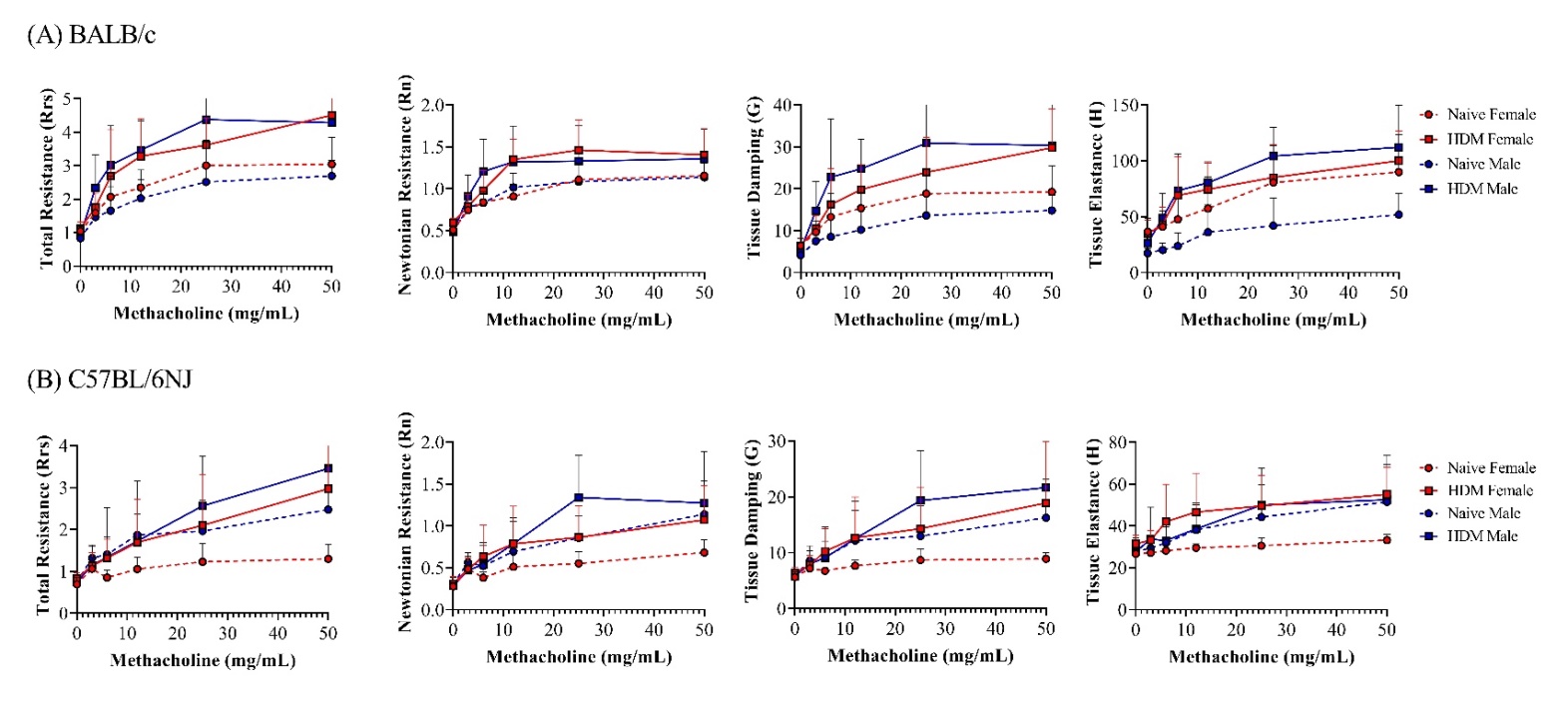


**Supplementary Figure 2: Cellular composition of BALF in BALB/c and C57BL/6NJ mice. (A)** BALB/c mice (n=9 each, female and male, per group) and **(B)** C57BL/6NJ (n=10 each, female and male, per group) were challenged (i.n) with 35 μL of 0.7 mg/mL whole HDM protein extract in saline per mouse, once daily for 5 consecutive days with two day rest in between for 2 weeks. BALF was collected 24 h after the last HDM challenge, and cell differentials assessed with modified Wright-Giemsa stain. Data shown represents mean percentage of each cell type, with total leukocytes set to a 100 percent.


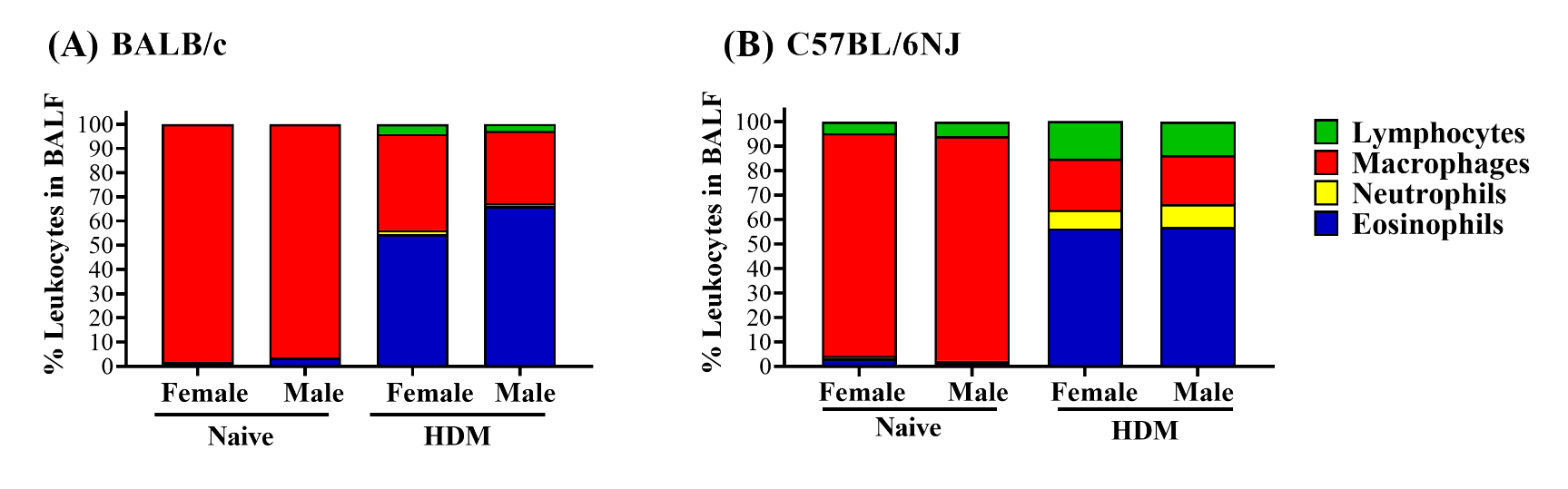


**Supplementary Figure 3: Leukocyte accumulation in BALF of BALB/c and C57BL/6NJ mice. (A)** BALB/c mice (n=9 each, female and male, per group) and **(B)** C57BL/6NJ (n=10 each, female and male, per group), were challenged (i.n) with 35 μL of 0.7 mg/mL whole HDM protein extract in saline per mouse, once daily for 5 consecutive days with two day rest in between for 2 weeks. BALF was collected 24 h after the last HDM challenge, and total cells and cell differentials were assessed in BALF. Two-way ANOVA with Tukey’s multiple comparisons test was used to determining statistical significance.


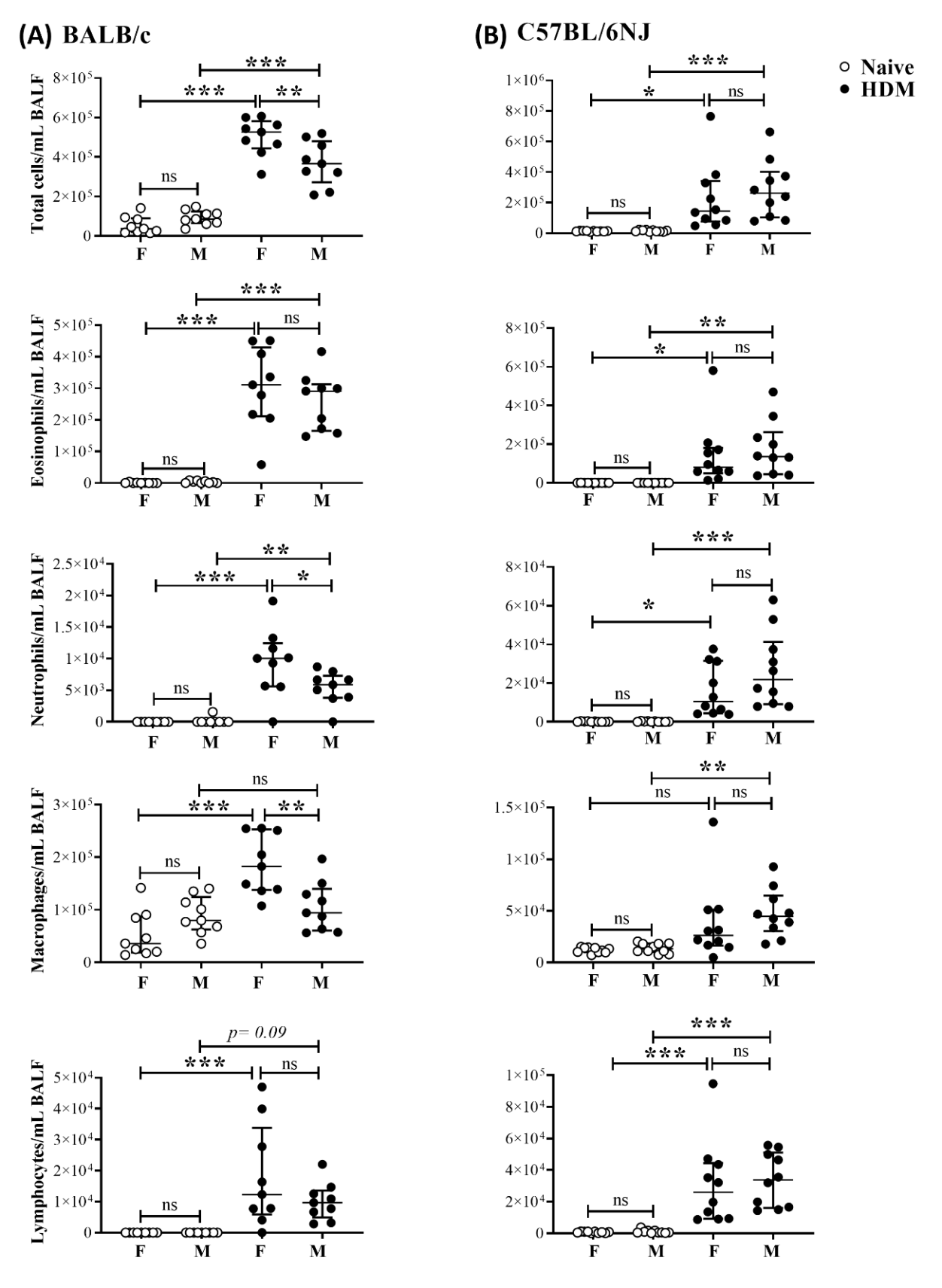


**(II) TABLES**

**Supplementary Table I.** **Cell differentials in BALF of HDM-challenged mice compared to allergen-naïve (HDM/naïve).**

|  | **BALB/c** | | | **C56BL/6NJ** | | |
| --- | --- | --- | --- | --- | --- | --- |
| **Cell Differentials** | **Female (F)**  **Ratio**  **HDM/naïve** | **Male (M)**  **Ratio**  **HDM/naïve** | ***p* value**  **(F vs M)** | **Female (F)**  **Ratio**  **HDM/naïve** | **Male (M)**  **Ratio**  **HDM/naïve** | ***p* value**  **(F vs M)** |
| **Total cells** | 8.0 | 3.0 | *p*<0.0001 | 17.0 | 19.0 | NS |
| **Eosinophils** | 472 | 60 | *p*<0.0001 | 351 | 789 | *p*<0.02 |
| **Neutrophils** | 9418 | 34 | *p*<0.003 | 110 | 165 | ns |
| **Macrophages** | 3.0 | 1.0 | *p*<0.0001 | 3.0 | 4.0 | ns |
| **Lymphocytes** | 18121 | 10028 | ns | 5331 | 72 | ns |

*ns = Non Significant*

**Supplementary Table II: Cytokine abundance in BALF in response to HDM-challenge.**

*ND=not detected*

| Cytokine | BALB/c | | | C57BL/6NJ | | |
| --- | --- | --- | --- | --- | --- | --- |
|  | **Female (F) HDM/naïve** | **Male (M) HDM/naïve** | ***p-value***  **(F vs M)** | **Female (F) HDM/naïve** | **Male (M) HDM/naïve** | ***p-value***  **(F vs M)** |
| IFNγ | 8.96 | 13.17 | *0.075* | 26.78 | 25.40 | *0.123* |
| IL-10 | 23.98 | 39.33 | *0.156* | 32.41 | 14.01 | *0.052* |
| IL-12p70 | 2.33 | 1.82 | *0.145* | 0.78 | 0.96 | *0.090* |
| IL-1β | 65.69 | 36.09 | *0.315* | 32.49 | 9.69 | ***0.034*** |
| IL-2 | 22.65 | 25.18 | *0.497* | 16.12 | 8.93 | *0.085* |
| IL-4 | 560.16 | 936.61 | *0.182* | 218.16 | 166.81 | *0.579* |
| IL-5 | 104.87 | 189.12 | *0.079* | 19.71 | 8.40 | *0.166* |
| IL-6 | 16.84 | 31.35 | *0.095* | 41.30 | 4.48 | *0.051* |
| KC/GRO | 7.90 | 5.88 | *0.549* | 9.20 | 4.23 | ***0.029*** |
| TNF | 2.20 | 3.16 | ***0.035*** | 17.94 | 10.50 | *0.143* |
| IL-16 | 38.60 | 31.60 | *0.604* | 12.11 | 6.44 | *0.089* |
| IL-17A | 156.01 | 8.28 | ***0.005*** | 406.53 | 262.95 | *0.841* |
| IL-17C | ND | ND | ND | ND | ND | ND |
| IL-25 | ND | ND | ND | ND | ND | ND |
| IL-17F | ND | ND | ND | ND | ND | ND |
| IL-21 | ND | ND | ND | ND | ND | ND |
| IL-22 | ND | ND | ND | ND | ND | ND |
| IL-23 | ND | ND | ND | ND | ND | ND |
| IL-31 | ND | ND | ND | ND | ND | ND |
| MIP3α | 1.47 | 1.19 | *0.589* | 9.74 | 8.62 | *0.166* |
| IL-15 | ND | ND | ND | ND | ND | ND |
| IL-17A/F | ND | ND | ND | ND | ND | ND |
| IL-30 | ND | ND | ND | ND | ND | ND |
| IL-33 | 3.01 | 6.76 | *0.243* | 1.09 | 0.45 | ***0.029*** |
| IL-9 | ND | ND | ND | ND | ND | ND |
| IP-10 | 3.91 | 5.86 | ***0.017*** | 7.59 | 3.72 | ***0.023*** |
| MCP1 | 123.08 | 254.20 | ***0.002*** | 200.05 | 73.94 | *0.063* |
| MIP1α | 3.30 | 7.56 | ***0.006*** | 18.01 | 8.62 | *0.218* |
| MIP2 | 3.58 | 3.35 | *0.968* | 6.73 | 2.01 | ***0.002*** |

**Supplementary Table III: Cytokine abundance in lung tissue lysates in response to HDM-challenge.**

| Cytokine | BALB/c | | | C57BL/6NJ | | |
| --- | --- | --- | --- | --- | --- | --- |
|  | **Female (F) HDM/naïve** | **Male (M) HDM/naïve** | ***p-value***  **(F vs M)** | **Female (F) HDM/naïve** | **Male (M) HDM/naïve** | ***p-value***  **(F vs M)** |
| IFNγ | 6.69 | 6.76 | *0.905* | 5.78 | 7.26 | *0.579* |
| IL-10 | 13.35 | 15.23 | *0.968* | 10.85 | 6.82 | *0.123* |
| IL-12p70 | 1.00 | 1.17 | *0.065* | 1.65 | 1.42 | *0.138* |
| IL-1β | 9.96 | 9.06 | *0.720* | 7.17 | 3.80 | *0.123* |
| IL-2 | 7.60 | 11.40 | *0.315* | 13.44 | 28.21 | *0.684* |
| IL-4 | 50.10 | 61.85 | *0.549* | 70.09 | 55.72 | *0.393* |
| IL-5 | 27.55 | 34.00 | *0.278* | 15.89 | 11.98 | *0.529* |
| IL-6 | 6.33 | 5.10 | *0.842* | 8.38 | 3.71 | *0.166* |
| KC/GRO | 6.71 | 4.72 | *0.095* | 3.66 | 1.41 | ***0.012*** |
| TNF | 2.93 | 2.46 | *0.182* | 6.57 | 3.64 | *0.075* |
| IL-16 | 2.57 | 1.93 | *0.054* | 2.24 | 1.92 | *0.123* |
| IL-17A | 9.09 | 4.99 | ***0.028*** | 10.20 | 11.26 | *0.853* |
| IL-17C | 1.04 | 1.25 | *0.211* | 1.50 | 1.69 | *0.631* |
| IL-25 | 1.68 | 2.43 | ***0.028*** | 2.95 | 2.18 | *0.166* |
| IL-17F | 0.98 | 1.51 | *0.243* | 1.74 | 1.73 | *0.836* |
| IL-21 | 3.62 | 8.41 | ***0.0001*** | 24.46 | 6.14 | ***0.002*** |
| IL-22 | 1.45 | 1.08 | *0.314* | 2.86 | 2.39 | *0.796* |
| IL-23 | 1.26 | 0.96 | *0.211* | 1.73 | 1.94 | *0.305* |
| IL-31 | 1.64 | 1.97 | *0.549* | 3.25 | 2.53 | *0.075* |
| MIP3α | 5.95 | 2.77 | ***0.002*** | 31.76 | 27.28 | *0.579* |
| IL-15 | 5.23 | 1.13 | *0.106* | 7.45 | 4.20 | *0.424* |
| IL-17A/F | 1.86 | 1.09 | *0.243* | 4.42 | 2.57 | *0.578* |
| IL-30 | 3.33 | 1.96 | ***0.004*** | 3.18 | 2.31 | *0.143* |
| IL-33 | 4.86 | 6.14 | *0.211* | 3.39 | 3.15 | *0.796* |
| IL-9 | 0.92 | 0.65 | ***0.014*** | 1.29 | 4.68 | ***0.002*** |
| IP-10 | 5.22 | 4.08 | *0.400* | 4.94 | 3.90 | *0.248* |
| MCP1 | 3.11 | 2.99 | *0.780* | 2.57 | 1.51 | *0.052* |
| MIP1a | 4.02 | 3.97 | *0.905* | 4.53 | 4.17 | *0.631* |
| MIP2 | 5.99 | 4.98 | *0.447* | 4.77 | 1.88 | ***0.003*** |
